# Eosinophilic peritonitis with colon cancer: a case report

**DOI:** 10.1186/s12876-020-01500-y

**Published:** 2020-10-27

**Authors:** Ryo Ataka, Hirokazu Tanaka, Shintaro Yagi, Kei Yamane, Kenji Yoshino, Tomoyuki Miyauchi, Tomoaki Yoh, Keiichi Arafuka, Shinichi Fujita, Akihiko Hamada, Bunji Endo, Shinji Uemoto

**Affiliations:** 1 Department of General Surgery, Tango Central Hospital, 158-1 Sugitani, Mineyama-Cho, Tango, Kyoto, 627-8555 Japan; 2 Department of Surgery, Graduate School of Medicine, Kyoto University, Kyoto, Japan; 3 Department of Gastroenterology, Tango Central Hospital, Kyoto, Japan

**Keywords:** Eosinophilic peritonitis, EGIDs, Colon cancer, Laparoscopy

## Abstract

**Background:**

Eosinophilic gastrointestinal disorders (EGIDs) are a rare group of inflammatory disorders that can occur anywhere along the gastrointestinal tract, from the esophagus to the rectum. In particular, those with malignant or benign tumors are extremely rare.

**Case presentation:**

A 62-year-old man was referred to our hospital with a chief complaint of abdominal fullness. The peripheral white blood cell count was 19,400/µL, and the eosinophil count was 13,300/µL. Abdominal computed tomography showed massive ascites. Cytology of the ascitic fluid showed a large amount of eosinophils and no malignancy. Upper and lower gastrointestinal endoscopies were performed on the suspicion of EGIDs, and colon cancer with no other abnormalities was found. The biopsies of the cancer lesions and non-cancer lesions also showed significant differences in eosinophil counts per high-power field (HPF) between the cancer and non-cancer lesions (median 77.5 [IQR 52–115] vs. 40.5 [35–56]/HPF, *P* < 0.05). Exploratory laparoscopy showed cloudy massive ascites and thickening of the mesentery. Pathological examination of the mesentery showed a large amount of eosinophils (median 177.5 [IQR 91–227]/HPF) and no malignancy. Based on these findings, it was suspected that the massive ascites due to eosinophilic peritonitis could be associated with colon cancer. Steroid administration resulted in immediate disappearance of the ascites, and laparoscopic left hemicolectomy was safely performed 6 weeks after steroid administration.

**Conclusion:**

This report presented a case of eosinophilic peritonitis that could be related to colon cancer. Exploratory laparoscopy was useful to detect the cause of ascites. The possibility that eosinophilic peritonitis was associated with colon cancer is discussed based on the histopathological findings.

## Background

Eosinophilic gastrointestinal disorders (EGIDs) are a rare group of inflammatory disorders that can occur anywhere along the gastrointestinal (GI) tract, from the esophagus to the rectum. In this group, eosinophilic esophagitis is the most common, and eosinophilic gastroenteritis and colitis are rarer [1]. Of them, EGIDs with malignant or benign tumors are extremely rare, with only four cases reported worldwide [2–5]. A case of colon cancer with massive ascites due to eosinophilic peritonitis, one subtype of the EGIDs, is presented.

## Case presentation

**Fig. 1:**
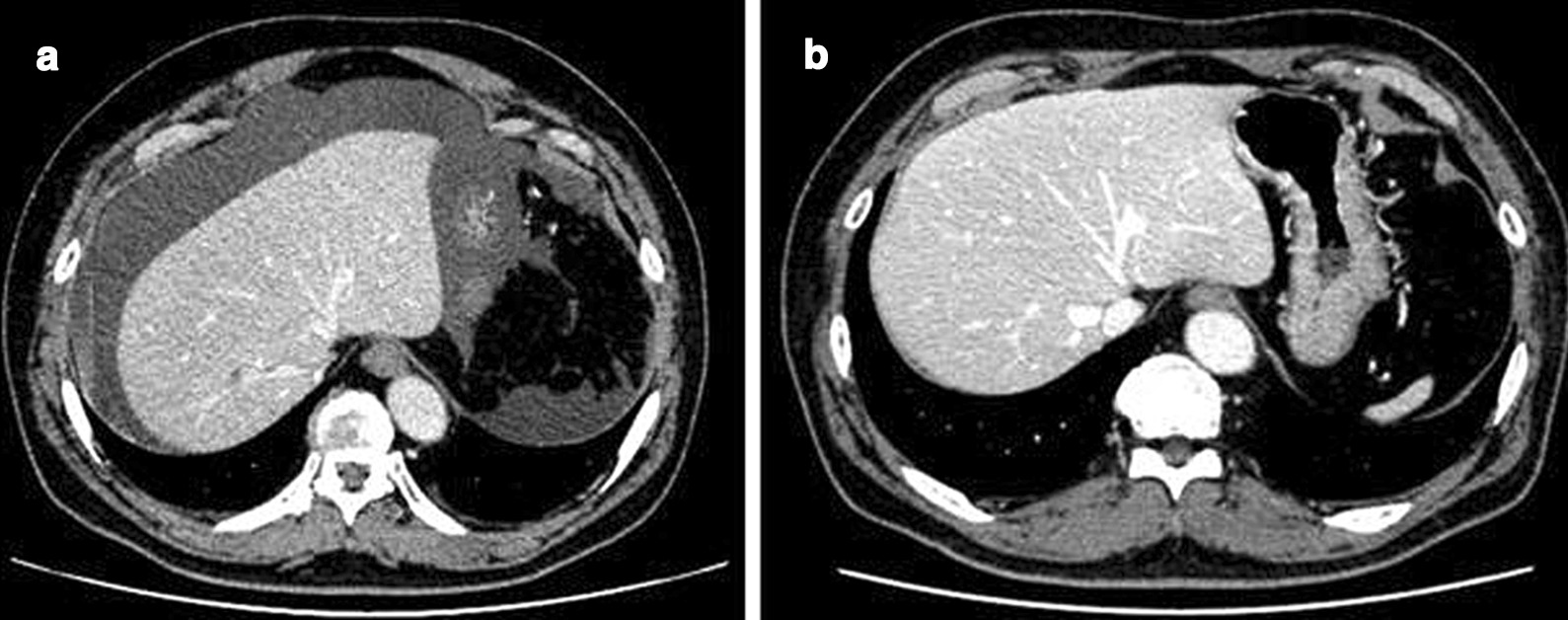
Abdominal CT with contrast. (**a**) Before steroid administration. (**b**) One and a half months after steroid administration

A 62-year-old man presented to a nearby clinic with a chief complaint of abdominal fullness and abdominal pain. His medical history was not significant. He was on no medication. He was not allergic to any drug or food. Laboratory tests showed increased peripheral white blood cell count (17,900/µL) and eosinophil count (12,100/µL), measured by an automated hematology analyzer, Sysmex XN 2000 (Sysmex corporation, Hyogo, Japan). Abdominal computed tomography (CT) showed massive ascites and no other abnormalities (Fig. 1a).

**Fig. 2:**
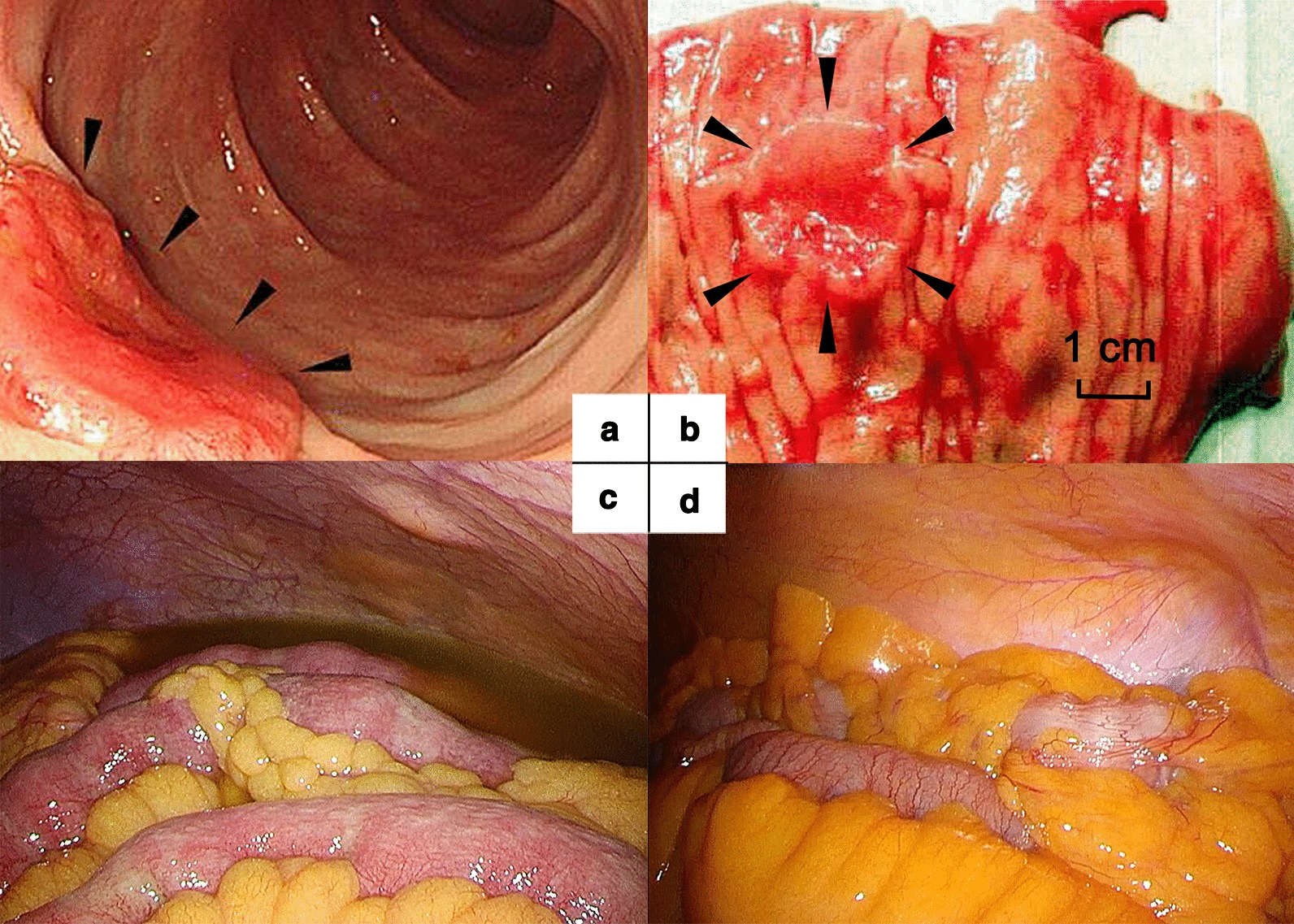
(**a**) Colonoscopy shows an abnormal reddish elevated lesion with excavation, suggesting colon cancer (arrow head). (**b**) Surgical specimen of the transverse colon involves a colon cancer lesion (arrow head). (**c**) First laparoscopy shows massive cloudy ascites. (**d**) Second laparoscopy shows disappearance of ascites

He was referred to our hospital for further examination and treatment. His height was 173 cm, his weight was 105 kg, and his body mass index was 35 kg/m^2^. He had gained 15 kg in the last month. Physical examination showed abdominal distention and no abdominal tenderness. The white blood cell count was 19,400/µL, eosinophil count was 13,300/µL, and C-reactive protein was 0.70 mg/dL. The other data were normal: carcinoembryonic antigen 3 ng/mL, carbohydrate 19–9 6 U/mL, and serum IgE 112 IU/mL. His allergen-specific IgE tests were unremarkable. The interferon-gamma release assay was negative. Abdominal magnetic resonance imaging (MRI) showed massive ascites that had low and high signal intensities on T1-weighted and T2-weighted images, respectively. Abdominal paracentesis was performed, and the ascitic fluid was stained with Fluoocell WDF (Sysmex corporation, Hyogo, Japan), then analyzed by flow cytometry with the Sysmex XN 2000 [6]. The total number of nucleated cells in the ascites was 11,420/µL, and 69% of them were eosinophils. Its cytology also revealed the most nucleated cells had both of bilobed nuclei and cytoplasmic granules, with no evidence of malignancy (Additional file 1: Figure S1). Ascitic fluid culture was negative. Upper and lower GI endoscopies were performed on the suspicion of EGIDs. There was a Type II-like lesion in the descending colon that showed well-differentiated adenocarcinoma on pathological examination of a biopsy specimen (Fig. 2a). There were no other abnormal findings in the GI tract. The patient underwent exploratory laparoscopy to find the cause of the ascites, and massive brownish cloudy ascites with reddening or thickening of the mesentery and omentum was seen. The tumor was not exposed to the colonic serosa. There were no enlarged lymph nodes, no abnormal adhesions, and no metastases (Fig. 2c). Pathology of the mesentery and omentum showed infiltration of inflammatory cells, mainly eosinophils, with no malignancy (Additional file 2: Figure S2). Based on these findings, eosinophilic peritonitis associated with colon cancer was suspected.

After administration of systemic steroid therapy (prednisolone (PSL) 60 mg/day, 1 mg/kg/day), his blood eosinophil count and weight decreased immediately. The dose of PSL was then decreased biweekly. One and a half months after steroid administration, abdominal CT confirmed disappearance of ascites (Fig. 1b). Laparoscopic left hemicolectomy with regional lymph node dissection (D3) was performed. At laparoscopy, the inflammation of the mesentery and omentum was seen to be relieved, and there was no ascites (Fig. 2b, d). The patient’s postoperative course was uneventful, and he was discharged on the 5th postoperative day. According to the Union for International Cancer Control TNM classification 8th Edition, the pathological diagnosis was tubular adenocarcinoma, moderately differentiated, pT3 (SS), int, ly1, v1, PN1, EX0, pN0, sM0, pStage IIA. The pathological specimen also showed eosinophilic infiltration to various lesions, especially omentum, mesentery, and the submucosal layer of the descending colon. He was given adjuvant oral chemotherapy (uracil-tegafur with leucovorin) for six months because there was concern that the pathological ly1 and v1 could increase the risk for recurrence of the cancer [7].

**Fig. 3:**
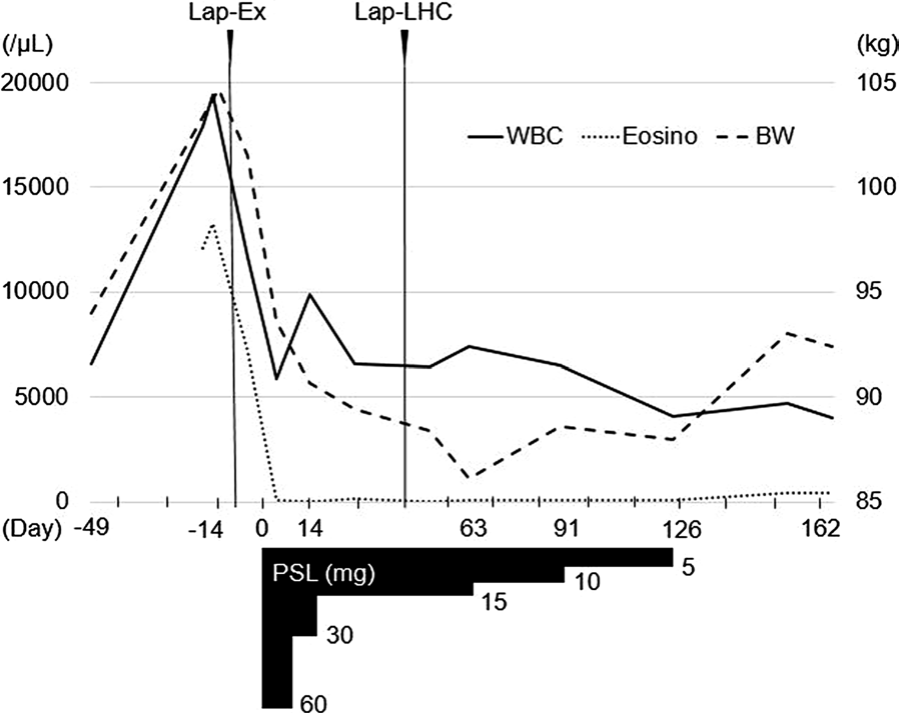
The clinical course of this case. Day 0 is the first day of steroid administration. The blood cell counts were determined by an automated hematology analyzer, Sysmex XN 2000, using flow cytometry technique with a semiconductor laser. Lap-Ex: exploratory laparoscopy. Lap-LHC: laparoscopic left hemicolectomy. PSL: prednisolone. WBC: white blood cell count (/µL). Eosino: Eosinophil count (/µL). BW: weight (kg)

After the operation, the dose of PSL was again decreased monthly. He finally stopped taking PSL four months after it was started, and he remains asymptomatic without recurrent ascites at the present time (Fig. 3).

## Discussion

Since Kaijser reported eosinophilic gastroenteritis for the first time in 1937 [8], EGIDs have been widely reported as a rare group of inflammatory GI diseases. In this group, eosinophilic gastroenteritis and colitis are rarer than eosinophilic esophagitis [1]. Mansoor et al. estimated that the prevalence of eosinophilic gastroenteritis was 5.1/100,000 persons, and that of eosinophilic colitis was 2.1/100,000 persons [9]. EGIDs present mainly in the third and fourth decades of life and are more common in men [10]. They are defined as inflammation with a characteristic eosinophilic infiltration into GI tract walls, in which various layers can be affected. Klein et al. in 1970 classified this disease as three subtypes, based on the depth of eosinophilic inflammation within the GI tract: A) predominant mucosal type (88%), in which eosinophils mainly infiltrate to mucosal layers, can cause vomiting, diarrhea, abdominal pain, GI bleeding, and protein-losing enteropathy; B) predominant muscle layer type (5.1%), in which eosinophils mainly infiltrate to muscle layers, often showing hypertrophy of GI walls, GI obstruction, and perforation; and C) predominant subserosal type (6.8%), in which eosinophils mainly infiltrate to subserosal layers, and can present with peritoneal hypertrophy and ascites. The third subtype is often called eosinophilic peritonitis [10, 11].

The diagnosis of EGIDs requires the (1) presence of recurrent GI symptoms, (2) demonstration of GI eosinophilic infiltration, and (3) absence of other causes of both. Although there is consensus on these diagnostic criteria for eosinophilic esophagitis, they are not fully applicable for other EGIDs because of the rarity and variations of these conditions [1]. EGIDs have been reported to have many differential diagnoses, such as drug allergy, food allergy, infection, tuberculosis, hypereosinophilic syndrome, autoimmune disease, inflammatory bowel disease, malignancy, graft-vs-host disease, and dialysis [1].

As for the treatment of EGIDs, systemic steroid therapy seems to be highly effective, with a clinical response in several months. In particular, predominant subserosal type appears to have the best response to steroid therapy of the three types [10]. Steroid therapy can also be more effective when used in combination with another therapy [11].

Among such rare diseases, few cases of EGIDs with malignant or benign tumors have been reported. In a literature search using PubMed for articles containing the keywords “eosinophilic enteritis” AND “tumor” OR “cancer” OR “malignancy”, it was found that, to date, there have been only five reported cases of EGIDs with malignant or benign tumors in the world, including the present case (Table 1) [2–5]. Of the five reported cases, three were the predominant mucosal type. This is the first reported case of predominant subserosal type with colon cancer.

**Table 1:** Reported cases of EGIDs with malignant or benign tumors

References	Age	Sex	Tumor	Subtype
Our case	62	M	Colon cancer	Predominant subserosal type
Otowa et al. [2]	69	M	Gastric cancer	Predominant mucosal type
Hui et al. [3]	45	F	Uterine leiomyomas	Predominant mucosal type
Stefanini et al. [4]	39	M	Large-cell anaplastic lung carcinoma	Predominant mucosal type
Ortega et al. [5]	N/A	N/A	Colon cancer	N/A

*N/A* not available

The current patient presented with a chief complaint of abdominal fullness due to ascites of unknown origin. Based on the peripheral eosinophilia and ascites cytology, EGIDs were most likely. The possibility of infection and other causes of peripheral eosinophilia seemed to be lower because of his past and present history, physical findings, and laboratory data. The diagnosis of eosinophilic peritonitis was finally made as the cause of the mysterious ascites by exploratory laparoscopy. As shown in this case, minimally invasive direct observation such as laparoscopy or natural orifice transluminal endoscopic surgery (NOTES) appears to be a valuable and effective approach to determine the cause of diseases with ascites of unknown origin [12].

This case of eosinophilic peritonitis is very unique given the presence of colon cancer, as mentioned above. There may be a certain relationship between eosinophilic peritonitis and colon cancer for the following several reasons.

**Fig. 4:**
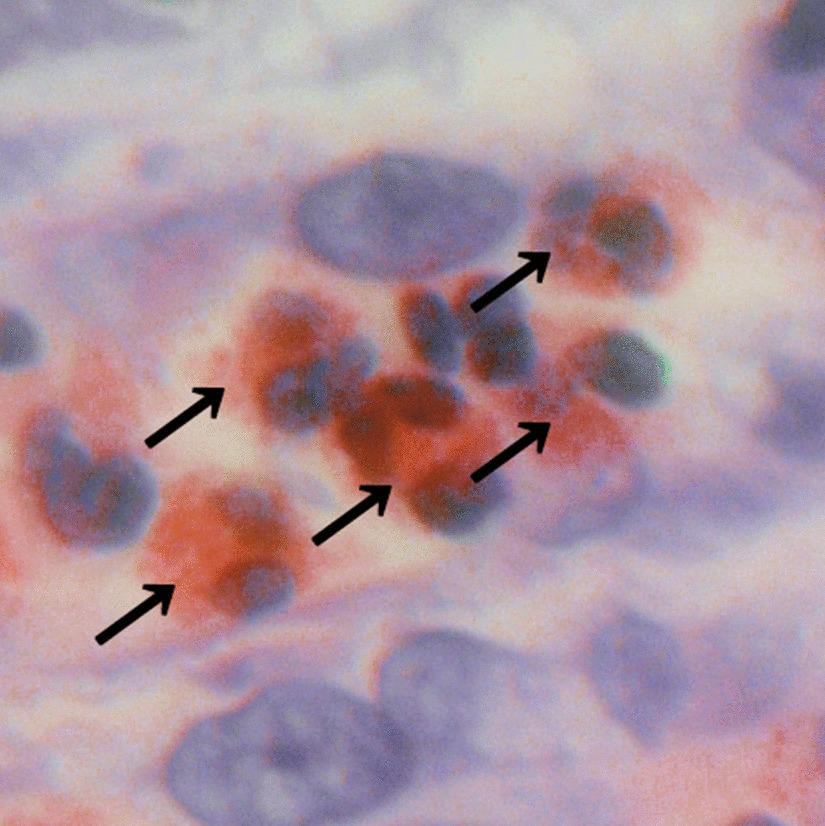
A pathological picture of cells around the colon cancer before steroid therapy (100 × objective and 10 × ocular). Eosinophils have bright red granules within their cellular cytoplasm and a nucleus with one or two lobes (black arrows)

**Fig. 5:**
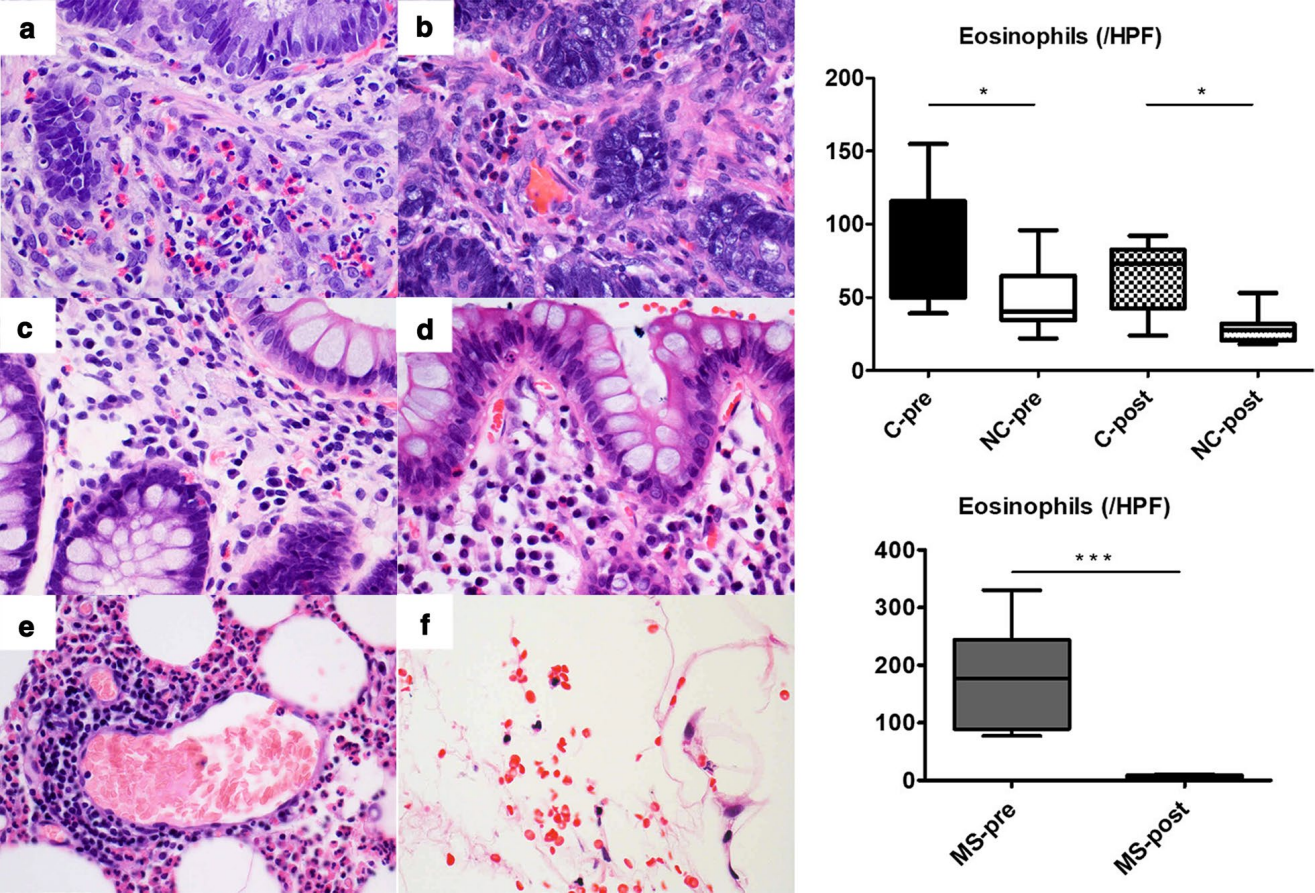
Six different pathological specimens (hematoxylin–eosin, 40 × objective and 10 × ocular), colon-cancer, non-cancer, and mesentery before steroid administration (A: C-pre, C: NC-pre, E: MS-pre), and colon-cancer, non-cancer, and mesentery after steroid administration (B: C-post, D: NC-post, F: MS-post). The graph above shows the eosinophil count per HPF in 20 randomly chosen HPFs. Data are medians and interquartile ranges. ^⋆^*P* < 0.05, one-way analysis of variance and Bonferroni’s multiple comparison test. The graph below shows the eosinophil count per HPF in 20 randomly chosen HPFs. Data are medians and interquartile ranges. ^⋆⋆⋆^*P* < 0.0001, *t*-test

The eosinophilic infiltration was compared between cancer and non-cancer lesions with a microscope ECLIPSE 80i (Nikon corporation, Tokyo, Japan), whose microscopic area was 0.237 mm^2^ per HPF (22 Field Number, 40 × objective and 10 × ocular). Hematoxylin–eosin staining of the specimens was performed for determination of cell distribution and morphology with the help of pathology technicians in our hospital. An eosinophil was identified as a colored cell with bright red granules within its cellular cytoplasm and a nucleus with one or two lobes (Fig. 4). Eosinophils were randomly counted in 20 high-power fields (HPFs) of 6 different histopathological lesions, cancer, non-cancer, and mesentery before steroid administration (C-pre, NC-pre, MS-pre), and cancer, non-cancer, and mesentery after steroid administration (C-post, NC-post, MS-post), shown in Fig. 5.

There were significant differences between C-pre and NC-pre (median 77.5 [IQR 52–115] vs. 40.5 [35–56]/HPF, *P* < 0.05), and between C-post and NC-post (median 73 [IQR 45–80] vs. 27.5 [21–30]/HPF, *P* < 0.05). Furthermore, there was a significant difference between MS-pre and MS-post (median 177.5 [IQR 91–227] vs. 6.5 [4–9]/HPF, *P* < 0.0001), while there was no significant difference between C-pre and C-post (median 77.5 [IQR 52–115] vs 73 [45–80]). These data suggest that there were more eosinophils around cancer lesions than around non-cancer lesions, both before and after steroid administration, and the systemic steroid therapy was completely effective to reduce mesenteric inflammation and ascites. However, these data also show that the eosinophilic infiltration still remained around the cancer lesions after steroid therapy.

Based on these results, one could hypothesize that the colon cancer may have been an antigen or allergen that stimulated eosinophil migration and induced eosinophilic infiltration. Moreover, colon cancer could have such strong effects on the eosinophilic infiltration around itself that the steroid therapy alone failed to completely resolve the phenomenon, and recurrence of peritonitis could develop. Furthermore, the surgical excision of the cancer could lead to the complete removal of the original stimulus for eosinophilic infiltration, and then steroid therapy could be stopped after surgery.

Some authors reported that colorectal cancer could have eosinophilic infiltration [13, 14], and that colorectal cancer had the potential for expression of various chemokines, such as interleukin (IL)-2, IL-3, IL-5, CCL (CC chemokine ligand) 11, or CCL24, that trigger eosinophil development and migration [15–17]. One of the limitations of this single case study is that the immunohistochemical expression and localization of those chemokines were not investigated. More similar cases are needed to obtain unknown pathological factors of eosinophilia in malignant tumors. In summary, given that the patient had no history of allergy, the actual eosinophilic distributions, and the unique clinical course, the present case may suggest the possibility of an association between eosinophilic peritonitis and colon cancer [2–4, 18].

The present patient received systemic steroid therapy for two months before surgery. In this case, comparing the size and shape of the tumor before and after steroid administration, the steroid therapy for a short period of time did not adversely affect the tumor. It actually had a positive effect on his general condition and surgical procedure. However, which should be done first, systemic steroid therapy or surgery, may be controversial.

## Conclusion

In conclusion, this report described the first case of eosinophilic peritonitis with colon cancer. This case showed the usefulness of exploratory laparoscopy as an effective way to detect the cause of mysterious ascites. This case also showed the possibility that eosinophilic peritonitis was associated with colon cancer.

## Supplementary information

**Additional file 1: Figure S1.** A cytological picture of the ascites with Papanicolaou staining (100 × objective and 10 × ocular). The most nucleated cells had bilobed nuclei and cytoplasmic granules. Furthermore, PAS staining, Giemsa staining and Papanicolaou staining were performed (not shown) and diagnosed that these nucleated cells were eosinophils.

**Additional file 2: Figure S2.** Leukocytes in the omentum with Hematoxylin-eosin staining (100 × objective and 10 × ocular). Eosinophils were distinguished from other leukocytes by both of bilobed nuclei and cytoplasmic granules (black arrows).

## Data Availability

Not applicable.

## Abbreviations

EGIDs: Eosinophilic gastrointestinal disorders
HPF: High-power field
GI: Gastrointestinal
CT: Computed tomography
MRI: Magnetic resonance imaging
PSL: Prednisolone
NOTES: Natural orifice transluminal endoscopic surgery
IL: Interleukin
CCL: CC chemokine ligand

## References

[1] Egan M, Furuta GT (2018). Eosinophilic gastrointestinal diseases beyond eosinophilic esophagitis. Ann Allergy Asthma Immunol.

[2] Otowa Y, Mitsutsuji M, Urade T (2012). Eosinophilic gastroenteritis associated with multiple gastric cancer. Eur J Gastroenterol Hepaol.

[3] Hui CK (2011). Resolution of eosinophilic gastroenteritis after resection of uterine leiomyomas. Singapore Med J.

[4] Stefanini GF, Addolorato G, Marsigli L (1994). Eosinophilic gastroenteritis in a patient with large-cell anaplastic lung carcinoma: a paraneoplastic syndrome?. Ital J Gastroenterol.

[5] Ortega G, Vidal JB, Molina M (1985). Eosinophilic gastroenteritis with extraintestinal involvement and cancer of the colon. Rev Clin Esp.

[6] Aguadero V, Cano-Corres R, Berlanga E (2018). Evaluation of biological fluid analysis using the sysmex XN automatic hematology analyzer. Cytometry B Clin Cytom.

[7] Schmoll HJ, Van Cutsem E, Stein A (2012). ESMO Consensus Guidelines for management of patients with colon and rectal cancer. a personalized approach to clinical decision making. Ann Oncol.

[8] Kaijser R (1937). Zur Kenntnis der allegishen Affektioner desima Verdauungskanal von Standpunkt desima Chirurgen aus. Arch Klin Chir.

[9] Mansoor E, Saleh MA, Cooper GS (2017). Prevalence of eosinophilic gastroenteritis and colitis in a population-based study, from 2012 to 2017. Clin Gastroenterol Hepatol.

[10] Talley NJ, Shorter RG, Philips SF (1990). Eosinophilic gastroenteritis: a clinicopathological study of patients with disease of the mucosa, muscle layer, and subserosal tissues. Gut.

[11] Chang JY, Choung RS, Lee RM (2010). A shift in the clinical spectrum of eosinophilic gastroenteritis toward the mucosal disease type. Clin Gastroenterol Hepatol.

[12] Bai Y, Qiao WG, Zhu HM (2014). Role of transgastric natural orifice transluminal endoscopic surgery in the diagnosis of ascites of unknown origin (with videos). Gastrointest Endosc.

[13] Moezzi J, Gopalswamy N, Haas RJ (2000). Stromal eosinophilia in colonic epithelial neoplasms. Am J Gastroenterol.

[14] Prizment AE, Anderson KE, Visvanathan K (2011). **I**nverse association of eosinophil count with colorectal cancer incidence: atherosclerosis risk in communities study. Cancer Epidemiol Biomarkers Prev.

[15] Anagnostopoulos GK, Sakorafas GK, Kostopoulos P (2005). Disseminated colon cancer with severe peripheral blood eosinophilia and elevated serum levels of interleukine-2, interleukine-3, interleukine-5, and GM-CSF. J Surg Oncol.

[16] Kato H, Kohata K, Yamamoto J (2010). Extreme eosinophilia caused by interleukin-5-producing disseminated colon cancer. Int J Hematol.

[17] Cho H, Lim SJ, Won KY (2016). Eosinophils in colorectal neoplasms associated with expression of CCL11 and CCL24. J Pathol Transl Med.

[18] Buka NJ (1965). Eosinophilia associated with uterine leiomyomas. Can Med Assoc J.

