# Supplementary figures and images for: Eosinophilic peritonitis with colon cancer: a case report
Source: BMC Gastroenterol. 2020 Oct 27;20:353. doi: 10.1186/s12876-020-01500-y (PMC7590699; doi:10.1186/s12876-020-01500-y)

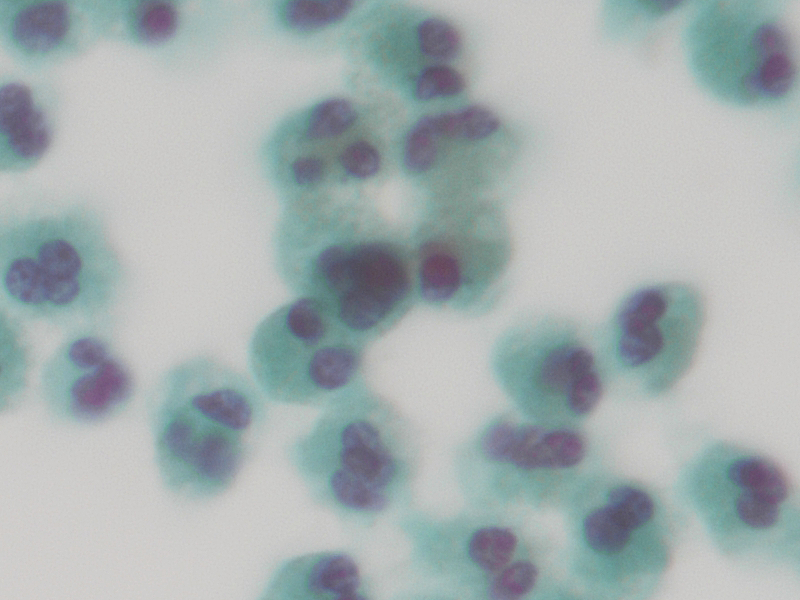

Supplement: Supplementary file 1 — Additional file 1: Figure S1. A cytological picture of the ascites with Papanicolaou staining (100 × objective and 10 × ocular). The most nucleated cells had bilobed nuclei and cytoplasmic granules. Furthermore, PAS staining, Giemsa staining and Papanicolaou staining were performed (not shown) and diagnosed that these nucleated cells were eosinophils. [file 12876_2020_1500_MOESM1_ESM.tif]

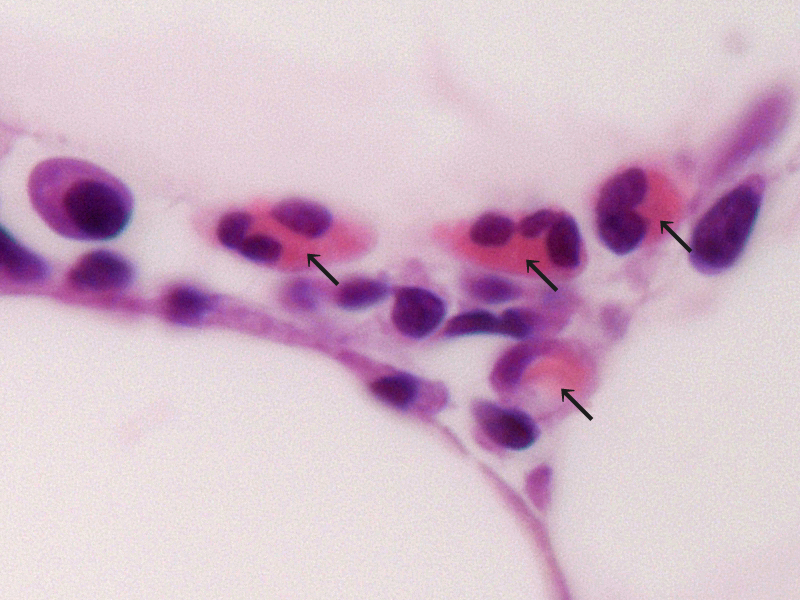

Supplement: Supplementary file 2 — Additional file 2: Figure S2. Leukocytes in the omentum with Hematoxylin-eosin staining (100 × objective and 10 × ocular). Eosinophils were distinguished from other leukocytes by both of bilobed nuclei and cytoplasmic granules (black arrows). [file 12876_2020_1500_MOESM2_ESM.tif]
